# Development of Body Weight Management Among the Hungarian Population

**DOI:** 10.3390/healthcare12222236

**Published:** 2024-11-10

**Authors:** Cintia Szilágyi, Mihály Soós, Marietta Kiss

**Affiliations:** 1 Doctoral School of Management and Business, Faculty of Economics and Business, University of Debrecen, H-4032 Debrecen, Hungary; 2 Institute of Marketing and Trade, Faculty of Economics and Business, University of Debrecen, H-4032 Debrecen, Hungary; soos.mihaly@econ.unideb.hu (M.S.); kiss.marietta@econ.unideb.hu (M.K.)

**Keywords:** body weight management, fad diet, obesity, health-conscious lifestyle, healthy eating, physical activity

## Abstract

Background/Objectives: Nowadays, body weight management has gained significant consumer attention due to the health megatrend. It plays a key role in preventing diseases, maintaining health, and creating a balanced diet, as well as in mental and physical health. Overweight and obesity are serious problems that can result in various non-communicable diseases; thus, managing the body weight of those who are overweight or obese without pathological changes would help reduce the prevalence of these diseases. By reducing body weight, not only individual health expenses but also public health costs could be reduced. To do so, it is necessary to assess at what levels consumers perceive their own health status, as well as the extent to which they pay attention to body weight management. Our research aimed to contribute to the foundation of public intervention programs by exploring some characteristics of body weight management among Hungarian consumers. Methods: We used a large-sample questionnaire survey involving a total of 550 people. Various statistical methods were used to analyze the data, including descriptive statistics, association tests, and hypothesis tests. Results: According to our results, those who want to lose weight are more engaged in managing their body weight than those who are satisfied with their weight or who want to gain weight, often aiming to reach an ideal weight through diet and exercise, and are willing to invest more in healthier lifestyles, both in terms of their diet and physical activity. In this regard, we did not find significant differences between the genders. Conclusions: Based on our findings, we can conclude that dietitians and doctors should play a more active role in guiding lifestyle changes since the majority of our respondents do not consider them credible sources of information on healthy eating, and they do not consult them on this topic. On the other hand, however, a much larger proportion seek information from them regarding regular physical activity, and after personal trainers, they are considered the second most authentic source of information.

## 1. Introduction

Obesity and its health and economic burden have been regarded as significant challenges over recent decades. Excess body weight without pathological alterations and non-communicable diseases appearing as complications generate problems all over the world, and Hungary is not an exception [1]. In Hungary, the number of overweight people has grown rapidly. The ‘thin’ category, on the other hand, has decreased, but not in each case in favor of those with a normal body mass index. Now, 68% of the Hungarian population is overweight or obese, which is the highest value in Europe, and globally the fifth behind only Mexico, the United States, Costa Rica, and New Zealand [2]. Being overweight may cause problems either mentally or physically; thus, in 2017, the WHO declared obesity officially to be a civilization disease that requires treatment [3,4]. Several preventive or health-maintaining methods, including nutritional and lifestyle changes, are already available, as well as drug treatment and surgical intervention to reduce body weight [5,6,7]. Until now, however, none of these have proven to be a breakthrough and general solution to the problem.

Health-conscious behavior and lifestyle have become trends nowadays. These include a healthy and balanced diet, regular and high-quality physical activity, and the prominent role of mental well-being [8,9]. Preventive programs impose a significant financial burden on healthcare systems, both at the individual and national level. In addition to adult obesity, an increasing trend of childhood obesity is typical not only in Hungary but also in Europe; at the same time, the number of years spent in healthy conditions among adults is significantly lower in Hungary compared to Europe [10,11,12,13]. It is, therefore, worth laying the foundations of a health-conscious lifestyle conveyed to children at an early age since being overweight can be extremely dangerous in childhood. For people with excess weight, the largest problems include not only social integration and the maintenance of their mental health but also the unfavorable health effects of the disease (including type 2 diabetes, heart disease, high blood pressure, certain cancers, etc.), which may appear even earlier due to being overweight for a long time. Therefore, the number of disease-free years is reduced, which many associate with the concept of health [14,15,16,17]. Törőcsik [18] defined several health-conscious food consumption options, including functional, ‘healing’, health and wellness, organic, ‘free-from’, and plant-based foods.

For today’s consumers, nutrition represents a form of loyalty. Especially in Generation Z (those born between 1995 and 2009), there is a phenomenon that lifestyle is not only a kind of individual trend but also reflects identity and fosters group formation among young adults [19]. Groups can be formed based on various goals, applied slimming diets and meal plans, nutritional trends arising from cultural differences, and similarities in food allergies and intolerances. Consumers can search for and share information about their own nutritional experiences in smaller groups within their personal environment while in larger groups online [20,21,22]. However, they do not always verify the credibility of the information exchanged, and, in most cases, when gathering nutrition-related information, they do not consider that the guidelines received in relation to a given dietary trend might carry potential dangers, as recommendations by blog posts, vloggers, and ‘self-proclaimed experts’ may not necessarily be based on scientific research results [23,24]. For instance, the most popular weight loss diets in 2021 in Hungary were the 160-g diet, carbohydrate-free diet, and ketogenic diet; however, they can cause quite a few health issues if applied inappropriately [25]. The problem with this approach is if someone is not distinguished based on whether they are healthy and simply follow a nutritional trend for fashion or if they suffer from an existing disease, such as lactose intolerance or gluten sensitivity. If a consumer with such a condition follows advice that does not meet the needs of his or her body, it may lead to a medical problem with a more serious outcome. However, most people start such kinds of diets due to excess weight or dissatisfaction with their weight and personal attributes. However, these diets are typically followed temporarily as part of a short-term regimen. Nutrition that can be sustained in the long term must be associated with lifestyle changes to achieve the desired goal [26,27,28].

The transition to a healthy lifestyle, including healthy eating, however, is not an automatic process but rather requires a long period, as individuals go through several stages to reach sustainable health behavior. The so-called Transtheoretical Model (TTM) is the most popular model for defining the stages of health behavior change. The model assesses individuals’ willingness to adopt a new, perceived healthy behavior through five stages [29,30]:

- Precontemplation: The individual does not plan to change their behavior within the next 6 months;
- Contemplation: The individual is committed to changing their behavior within the next 6 months;
- Preparation: The individual is ready to take action in the near future (typically within the next month);
- Action: The behavior change began at least 6 months ago;
- Maintenance: The action has been ongoing for more than 6 months, and the chances of reverting to the old behavior are minimal.

The TTM is based on the assumption that an intervention is most effective when it aligns with the individual’s current stage. Horwath [31] applied the model specifically to assess changes in eating behavior.

**Figure 1:**
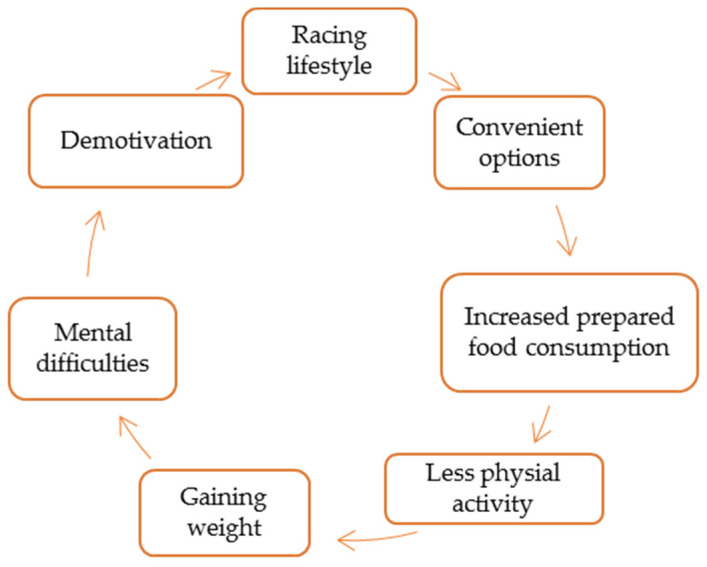
Average consumer cycle. Source: Authors’ illustration.

Eating behavior has been influenced by several external factors in recent years, such as the pandemic, the war, the increasing Internet penetration, and the rapid development of smart devices. Consequently, it is not surprising that the focus of most studies related to self-control is nutrition and the resulting excess weight [32]. In summary, it can be said that a consumer is forced into a cycle, which is illustrated in Figure 1.

These days, a fast-paced lifestyle is becoming the norm. The increasing number of technical innovations and developments gives us the opportunity to satisfy all the comfort functions and needs even in our own homes with our own devices. As a result of the pandemic, the proportion of products (including food products and meals) ordered online also increased. Consumers have become accustomed to or—even dependent on—the ease and comfort of online shopping and similar conveniences, thus developing unhealthy lifestyles with decreasing physical activity, which may result in being overweight. Recognizing this process is easier for consumers, yet they do not always take steps to change. As seen in Maslow’s hierarchy, they seek safety when they reach complete mental burnout, and the only solace they find is immersing themselves in work. Thus, the consumer returns to a fast-paced and comfortable lifestyle, starting the vicious cycle all over again [33]. Of course, the process does not take place within a brief period in the individual’s life. They might fall into this cycle only once or, in worse cases, several times, and there is a possibility that they will not be able to get out of it. The main problem, however, is the processing and flow of information since an exceptionally significant percentage of those who lead a fast-paced lifestyle spend most of their lives on online social platforms, looking for solutions to their problems.

Based on these facts, we started our research to be able to help people get out of the cycle or prevent people from entering it, thereby reducing individual health problems and individual- and national-level illness costs. Our research aimed to contribute to the foundation of public intervention programs by exploring some characteristics of body weight management among Hungarian consumers. We aimed to examine the following hypotheses:

**H1.**  *The majority of those involved in weight management are women.*

**H2.**  *Body weight management is more commonly pursued by those who wish to lose weight than those who do not intend to lose weight.*

**H3.**  *The sources of information that consumers consider authentic for healthy nutrition differ from those they consider reliable for appropriate physical activity.*

## 2. Materials and Methods

The method of our primary data collection was a large-sample questionnaire survey in which 550 Hungarian adults were interviewed online on the topic. We shared the questionnaire on Facebook, thus enabling a snowball sampling method since participants were asked to share it among their friends on the platform. The questionnaire form was set to be limited to accept only one response per respondent. The time interval for completing the questionnaire was between 7 December 2022 and 1 January 2023.

The questionnaire contained 16 questions. First, we asked the respondents whether they were satisfied with their body weight or if they would like to lose or gain weight. In addition, we approximated involvement in body weight management using five variables: first, respondents were asked to rate on an 8-point Likert scale the extent to which they restrict themselves in their eating (1 = not at all, 8 = completely); then, they were asked to indicate their stage in the process of behavior change according to the TTM in terms of transitioning to healthier eating and regular physical activity; additionally, we asked whether they would be willing to spend more than they currently do on foods and physical activities that benefit their health (yes/no/don’t know). Finally, respondents had to indicate the information sources they use and consider the authenticity of these sources on healthy eating and regular physical activity (yes/no per information source). At the end of the questionnaire, socio-demographic questions were included (gender, age, education, and subjective income status, as well as height and weight for calculating BMI). To form groups of respondents based on their BMI, the WHO’s classification was used [34]. The survey questions can be found in Appendix A.

Various statistical methods were used to analyze the data, including descriptive statistics (frequencies, mean, and standard deviation), association tests (cross-tabulation analysis and Chi-square tests), and hypothesis tests (the Mann–Whitney U test, since the normality assumption was not met by the variable in question, and ANOVA with the Scheffe posthoc test) [35,36]. The analyses were carried out with the use of IBM SPSS 21 software.

## 3. Results

The distribution of the sample based on socio-demographic characteristics is shown in Table 1. Regarding gender, the sample is representative of the Hungarian population [37].

The average BMI of the participants in the survey was 23.2 (standard deviation: 4.28), with the lowest value being 15.7 and the highest 39.9. According to the WHO [34] classification, 11.8% of the respondents were underweight, 61.1% had a normal weight, 19.3% were overweight but not obese, and 7.8% were obese. Among men, 2.7% were underweight, 40.9% had a normal body weight, 40.2% were overweight, and 16.3% were obese; among female respondents, 20.3% were underweight, and 79.7% had a normal body weight based on the provided height and weight data. There is a significant, moderately strong relationship between the two variables, i.e., gender and BMI class (see Table 2). There is a statistically higher proportion of overweight and obese men in the sample than expected, as well as underweight and normal-weight women (see Table 3).

We also asked whether the respondents would like to change their body weight; that is, whether they would like to lose or gain weight or if they were satisfied with their current weight. The majority of the sample (53.8%) wanted to lose weight, and 38.2% were satisfied with their weight, while only 8.0% wanted to gain weight. We did not find a statistically significant difference in the intention to change body weight based on gender (see Table 2).

A comparison of the intention to change body weight and BMI revealed a significant but weak relationship between the two variables (see Table 2). Overweight and obese individuals were somewhat more likely to want to lose weight than statistically expected and somewhat less likely to want to gain weight. Nevertheless, 30.5% of the overweight and 27.9% of the obese individuals were satisfied with their body weight. A positive result is that underweight individuals were statistically more likely to want to gain weight and less likely to want to lose weight; however, it is somewhat concerning that they were more likely to be satisfied with their weight than statistically expected (see Table 4).

From the gender breakdown, it is revealed that men with a normal body weight are less likely to want to lose weight than statistically expected, while overweight and obese men are more likely. In the lower two BMI categories, men are more likely to want to gain weight than statistically expected, while in the upper two categories, they are less likely to want to gain weight. Men with a normal body weight are more likely to be satisfied with their weight than statistically expected, while overweight men are less likely to be satisfied (see Table 5). For women, underweight individuals are more likely to be satisfied with their weight and want to gain weight than statistically expected and less likely to want to lose weight. Women with a normal body weight are more likely to want to lose weight than statistically expected and less likely to be satisfied with their bodies or want to gain weight (see Table 6).

To examine the first (H1) and second (H2) hypotheses, we approximated involvement in body weight management using five variables. According to our results, the average level of dietary restriction (measured on an 8-point Likert scale) shows a numerical difference between the two genders (men: mean: 3.22, SD: 1.822; women: mean: 3.44, SD: 1.924), but this difference was not significant (U = 35,426.0, *p* = 0.205). Regarding the stages of transitioning to healthier eating according to the TTM, 21.6%, 22.4%, 17.3%, 18.7%, and 20.0% of respondents reported being in each of the five successive stages. By gender, we found a difference only at a 10% significance level, with a weak association (see Table 7); men were more likely than expected to be in the first stage of change (Precontemplation), while women were less likely (adjusted standardized residuals: 2.3 and −2.3, respectively). In line with our previous findings, we also found no significant gender difference in the willingness to spend more on health-promoting foods (see Table 7). Regarding the five stages of transitioning to regular physical activity according to the TTM, the distribution of respondents was 8.0%, 24.4%, 19.6%, 22.7%, and 25.3%, respectively. We found a significant gender difference, with a weak association (see Table 7); men were more likely than expected to be in the first and fifth stages (Precontemplation and Maintenance) and less likely to be in the third stage (Preparation), while women showed the opposite pattern (see Table 8). We found no significant gender difference in the willingness to spend more on health-promoting physical activity (see Table 7).

To test the second hypothesis (H2), we first examined whether there is a significant difference in the self-restriction of eating among those who want to lose weight, those who want to gain weight, and those who are satisfied with their bodies. Our results show that the three groups differ significantly in this regard (F(2,547) = 35.907, *p* < 0.001; Scheffe posthoc tests in all cases: *p* < 0.001), with the highest level of self-restriction observed among those who want to lose weight (mean: 3.83, SD: 1.722), followed by those who are satisfied with their weight (mean: 2.98, SD: 1.895), and the lowest level of control observed among those who want to gain weight (mean: 1.66, SD: 1.397). The three groups also differ significantly regarding the stages of transitioning to healthier eating according to the TTM, with a moderately strong association between the two variables (see Table 9). Those who want to lose weight are underrepresented in the first and fifth stages of the TTM and overrepresented in the other stages. Those who want to gain weight are overrepresented in the first stage, while those satisfied with their body weight are overrepresented in the first and fifth stages and underrepresented in the third and fourth stages (see Table 10). No significant difference was found between the three groups regarding their willingness to spend more on healthier foods (see Table 9); however, when excluding those who answered ‘don’t know,’ the differences were significant at the 10% significance level (Chi-square: 4.890, df = 2, *p* = 0.087), though weak (Cramer’s V: 0.102, *p* = 0.087). Those who want to lose weight were somewhat more willing to spend more on healthier foods than those who want to gain weight and those who are satisfied with their weight.

The three groups also differ significantly regarding the stages of transitioning to regular physical activity according to the TTM, with a weak association between the two variables (see Table 9). Similar to the transition to healthier eating, those who want to lose weight are underrepresented in the first and fifth stages of the TTM and overrepresented in the third and fourth stages. Those who want to gain weight are overrepresented in the first stage, while those satisfied with their body weight are overrepresented in the fifth stage and underrepresented in the third and fourth stages (see Table 11). Differences in the willingness to spend more on regular physical activity were found only at the 10% significance level, though the relationship is very weak (see Table 9). Those who are satisfied with their body weight are somewhat more likely than statistically expected to report that they would not spend more on regular physical activity.

**Figure 2:**
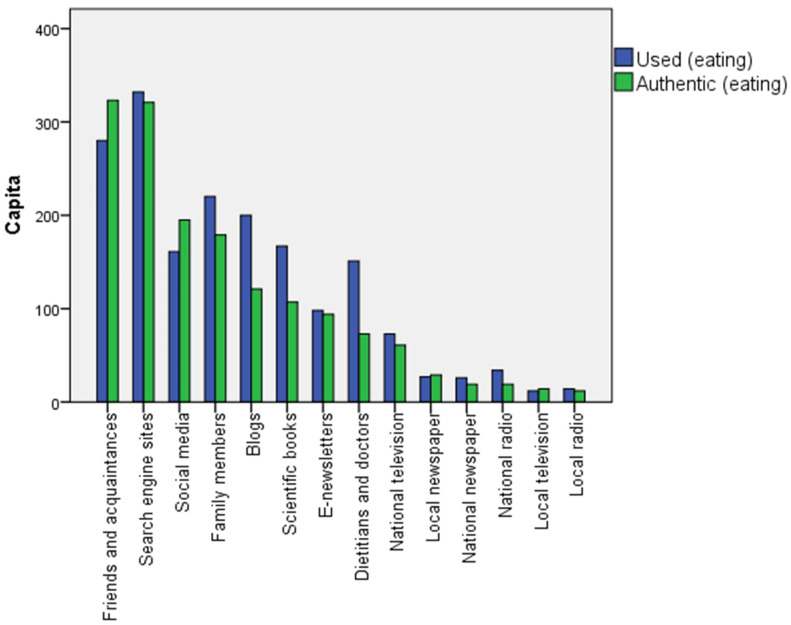
Information sources used and considered authentic by respondents regarding healthy nutrition in descending order according to authenticity (N = 550). Source: Authors’ compilation, 2024.

To examine our hypothesis regarding the sources of information (H3), we allowed respondents to mark several options. Friends and acquaintances lead the authenticity scale with 323 (58.7%) answers regarding what is believed to be healthy nutrition. They are followed by 321 answers (58.4%) for Internet search sites, 195 answers (35.5%) for social media, and 179 (32.5%) for family members (Figure 2). Interestingly, the most used sources on healthy eating are search engine sites (60.4%), friends and acquaintances (50.9%), family members (40.0%), and social media (29.3%). Dietitians and doctors are not considered very authentic, and accordingly, they are not used among the most popular information sources.

**Figure 3:**
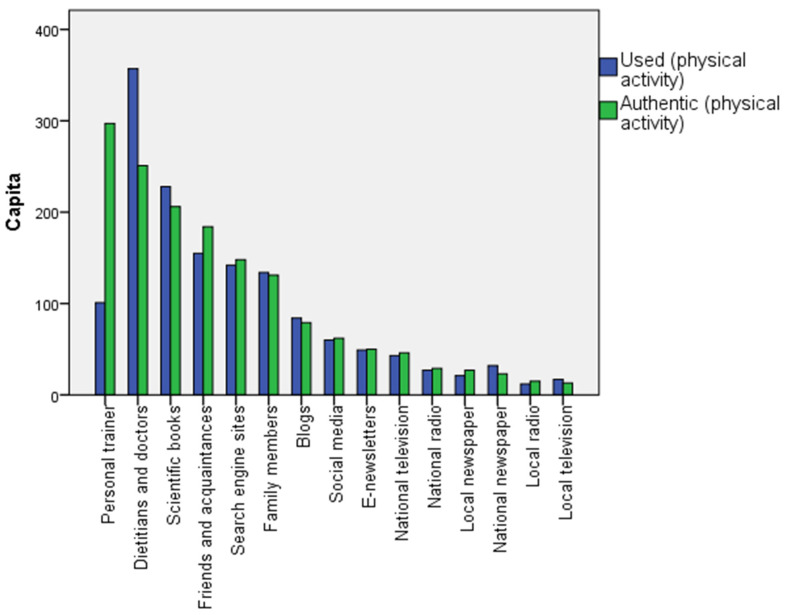
Information sources used and considered authentic by respondents regarding physical activities in descending order according to authenticity (N = 550). Source: Authors’ compilation, 2024.

As a source of information about physical activity, personal trainers were chosen as the most reliable 297 (54.0%) times, followed by dietitians and doctors with 251 (45.6%) answers, and scientific books with 206 (37.5%) answers as the top three authentic sources (Figure 3). The top information sources used on physical activities differ in their order of authenticity: most respondents use dietitians and doctors (64.9%), scientific books (41.5%), and friends and acquaintances (28.2%) to gather information on this topic.

## 4. Discussion

Nations are struggling with non-communicable diseases, of which excess weight is particularly important because, although it can be defined as a separate disease, it can also be a symptom of other serious diseases. Although the research on overweight individuals has a prominent role, nations that are still struggling with hunger and malnutrition cannot be neglected either. The problem of malnutrition or overnutrition should be addressed not only on an individual level but also on a national level to target consumers in danger with preventive programs and informative materials. Our research aimed to contribute to the foundation of these intervention programs by exploring some characteristics of body weight management among Hungarian consumers.

Per our expectations and previous research findings (e.g., [38,39]), the majority of respondents in our sample (nearly 54%) expressed a desire to lose weight to reach their perceived ideal body weight, while only a minimal proportion (8%) wanted to gain weight, with no significant gender differences observed. Similarly, as anticipated, the results indicate that overweight and obese individuals were somewhat more likely to want to lose weight; nevertheless, nearly one-third of the overweight and obese individuals were satisfied with their body weight. A positive result is that underweight individuals were more likely to want to gain and less likely to want to lose weight; however, it is somewhat concerning that they were more likely to be satisfied with their weight. The gender-based analysis reveals similar trends, except that women of normal weight were statistically more likely to want to lose weight than expected. This finding is not surprising, as several previous studies have reported that many women engage in weight loss efforts despite having a healthy BMI (see, e.g., [40,41]).

Our first hypothesis (H1) proposed that the majority of those involved in weight management are women. We approached body weight management through five variables, among which, despite some numerical differences suggesting higher female participation in weight management, only one variable showed a very weak significant difference (stage of transitioning to regular physical activity). Thus, overall, we conclude that there is no significant gender difference in this matter in our sample, and we reject the hypothesis. This result is surprising in light of previous research findings, as numerous studies have reported that women pay more attention to both healthy eating (see, e.g., [42]) and regular physical activity (see, e.g., [43]) than men, and actively seek information about these topics [42]. This result is likely due to the fact that our sample was not representative of the entire Hungarian population; for example, it did not reflect its BMI distribution.

According to our results, self-restriction in eating is highest among those who wish to lose weight and lowest among those who wish to gain weight. In the process of transitioning to healthier eating and regular physical activity, those who wish to lose weight have generally already started, while those who wish to gain weight typically do not plan to make any changes to their current lifestyle, though the relationship between the variables is weak to moderate. Those who wish to lose weight are also somewhat more willing to spend more on healthier foods and regular physical activity than those who do not wish to lose weight, though the relationship here is extremely weak. Based on these findings, we accept our second hypothesis (H2), which states that body weight management is more commonly addressed by those who wish to lose weight than by those who do not intend to lose weight. This result aligns with previous research indicating that weight loss goals significantly impact people’s engagement in various health behaviors, such as dietary changes and exercise. For instance, individuals motivated by weight loss are more likely to adopt strategies like reducing caloric intake, choosing healthier foods, and increasing physical activity compared to those who are not actively seeking weight loss [44,45].

According to our respondents, the most credible sources of information related to healthy eating are friends and acquaintances, Internet searches, social media, and family members, meaning that online and close personal sources dominate. These are also the most commonly used sources by the respondents (in different order). Unfortunately, dietitians and doctors are considered credible on the subject by significantly fewer individuals, and far fewer respondents use them to gather information, which contradicts the results of [46], according to which nutrition–health websites, Google–Internet searches, and diet–health books were most commonly used sources by the survey participants; however, they placed the highest level of trust in nutrition scientists, nutrition professionals, and scientific journals. The reasons for this contradiction may be multifaceted. Hungarian consumers may find that there is no time to ask for dietary advice in an overburdened healthcare system, or they may not even attempt to seek it. Sources of information regarding physical activity that are authentic are much closer to the sources recommended in the literature than those related to nutrition. Accordingly, the most credible source of information regarding regular physical activity is personal trainers [47], though dietitians and doctors also rank as the second most credible sources, followed by scientific books and friends and acquaintances. In terms of actions, however, dietitians and doctors stand out as the most common sources from whom individuals seek information, followed by scientific books and friends and acquaintances; the use of personal trainers is much less common, likely for financial reasons. This is in line with the findings of [46], according to which the use of an information source may not be a reliable predictor of the level of trust assigned to it.

The previous results draw attention to the need for more effective communication of the fact that not all information sources are considered authentic and scientifically based to consumers. Dietitians need to act in greater numbers on platforms where active consumers have started their lifestyle changes and where they need accurate guidance on their journey to health by developing the correct nutrition. The transformation of the communication and marketing strategies applied to those who have not yet started lifestyle changes must be given a priority role in the future since without a paradigm change or change in the transfer of a lifestyle based on traditions to the younger generations, a healthy nation that exists economically and environmentally in the long term cannot be achieved.

One major limitation of the present study is related to the sample, which was representative of the Hungarian population only in terms of gender. Therefore, a potential direction for future research could be to address this limitation by expanding the study to a larger, nationally or even internationally representative sample using a probability sampling method. Additionally, the study relies on a self-administered survey, which, while offering cost-effectiveness, flexibility in timing, and respondent privacy, also has drawbacks, such as potential bias in the respondents’ self-reported weights (although this distortion is likely lower online than it would be in a face-to-face interview). Thus, in future studies, collecting respondents’ actual weights could yield more reliable information on their BMI. Finally, in this research, we did not study the actual but only the perceived authenticity of information sources used by respondents and the reasons behind their choices of various information sources; however, this provides another interesting research direction.

## 5. Conclusions

Countries are grappling with non-communicable diseases, with excess weight being particularly significant. Addressing malnutrition and overnutrition requires both individual and international efforts, including preventive programs and educational materials. Our research shows those who want to lose weight are more engaged in managing their body weight than those who are satisfied with their weight or who want to gain weight, often aiming to reach an ideal weight through diet and exercise and are willing to invest more in healthier lifestyles, both in terms of their diet and physical activity. In this regard, we did not find differences between the genders, probably due to the sample, which was representative only in terms of the genders and not for, e.g., BMI.

Despite the known benefits of diet and exercise for achieving an ideal weight, our findings indicate a gap in consumer reliance on credible health sources. To improve public health, dietitians and doctors should play a more active role in guiding lifestyle changes since the majority of our respondents do not consider them a credible source of information on healthy eating, and they do not consult them on this topic. Recognizing the potential impact of trusted guidance on public health, the purpose of this pilot research was to lay the foundation for the following studies, in which a solution can be found to curb the misinformation flowing toward consumers and support those who have not yet started on the path to a healthy lifestyle, with a marketing strategy that includes information from credible sources and health preventive programs.

## Figures and Tables

**Table 1:** Sample distribution according to background variables (N = 550).

Socio-Demographic Variables	Capita	%
Gender		
Male	264	48.0
Female	286	52.0
Age group		
18–25 years	333	60.5
26–35 years	151	27.5
36–45 years	34	6.2
46–55 years	17	3.1
56 years and above	15	2.7
Highest educational attainment		
Eight-grade elementary school at most	3	0.5
Vocational or technical school	13	2.4
Matura examination	169	30.7
Higher education degree	365	66.4
Subjective income situation		
Can live well from it and can also save money	74	13.5
Enough to live on but can barely save any money	275	50.0
Can make ends meet but cannot save money	136	24.7
Sometimes find(s) it difficult to make ends meet	26	4.7
Regularly find(s) it difficult to make ends meet	5	0.9
Don’t know/No answer	34	6.2

Source: Authors’ compilation, 2023.

**Table 2:** Relationship between gender, BMI class, and intention to change body weight (N = 550).

	Chi-Square	df	*p*	Cramer’s V
Gender × BMI	231.363	3	<0.001	0.649
Gender × Intention to change	2.939	2	0.230	-
BMI × Intention to change	93.604	6	<0.001	0.292

Source: Authors’ compilation, 2024.

**Table 3:** Cross-tabulation of gender and BMI class (N = 550).

			BMI Class				Total
			Underweight	Normal	Overweight	Obese	
Gender	male	count	7	108	106	43	264
		% within gender	2.7%	40.9%	40.2%	16.3%	100.0%
		adjusted standardized residual	−6.4	−9.3	11.9	7.1	
	female	count	58	228	0	0	286
		% within gender	20.3%	79.7%	0.0%	0.0%	100.0%
		adjusted standardized residual	6.4	9.3	−11.9	−7.1	
Total		count	65	336	106	43	550
		% within gender	11.8%	61.1%	19.3%	7.8%	100.0%

Source: Authors’ compilation, 2024.

**Table 4:** Cross-tabulation of intention to change weight and BMI class for total sample (N = 550).

			BMI Class				Total
			Underweight	Normal	Overweight	Obese	
Intention to change	want to lose weight	count	5	187	73	31	296
		% within intention to change	1.7%	63.2%	24.7%	10.5%	100.0%
		adjusted standardized residual	−7.9	1.1	3.5	2.5	
	want to gain weight	count	19	24	1	0	44
		% within intention to change	43.2%	54.5%	2.3%	0.0%	100.0%
		adjusted standardized residual	6.7	−0.9	−3.0	−2.0	
	satisfied	count	41	125	32	12	210
		% within intention to change	19.5%	59.5%	15.2%	5.7%	100.0%
		adjusted standardized residual	4.4	−0.6	−1.9	−1.4	
Total		count	65	336	106	43	550
		% within gender	11.8%	61.1%	19.3%	7.8%	100.0%

Source: Authors’ compilation, 2024.

**Table 5:** Cross-tabulation of intention to change weight and BMI class: Males (N = 264).

			BMI Class				Total
			Underweight	Normal	Overweight	Obese	
Intention to change	want to lose weight	count	2	29	73	31	135
		% within intention to change	1.5%	21.5%	54.1%	23.0%	100.0%
		adjusted standardized residual	−1.2	−6.6	4.7	3.0	
	want to gain weight	count	4	21	1	0	26
		% within intention to change	15.4%	80.8%	3.8%	0.0%	100.0%
		adjusted standardized residual	4.3	4.4	−4.0	−2.4	
	satisfied	count	1	58	32	12	103
		% within intention to change	1.0%	56.3%	31.1%	11.7%	100.0%
		adjusted standardized residual	−1.4	4.1	−2.4	−1.6	
Total		count	7	108	106	43	264
		% within gender	2.7%	40.9%	40.2%	16.3%	100.0%

Source: Authors’ compilation, 2024.

**Table 6:** Cross-tabulation of intention to change weight and BMI class: Females (N = 286).

			BMI Class		Total
			Underweight	Normal	
Intention to change	want to lose weight	count	3	158	161
		% within intention to change	1.9%	98.1%	100.0%
		adjusted standardized residual	−8.8	8.8	
	want to gain weight	count	15	3	18
		% within intention to change	83.3%	16.7%	100.0%
		adjusted standardized residual	6.9	−6.9	
	satisfied	count	40	67	107
		% within intention to change	37.4%	62.6%	100.0%
		adjusted standardized residual	5.6	−5.6	
Total		count	58	228	286
		% within gender	20.3%	79.7%	100.0%

Source: Authors’ compilation, 2024.

**Table 7:** Relationship between gender and TTM stages toward healthy eating and regular physical activity and willingness to spend more on health-promoting foods and regular physical activity (N = 550).

	Chi-Square	df	*p*	Cramer’s V
Gender × TTM stages toward healthy eating	9.199	4	0.056	0.129
Gender × WTS more on health-promoting foods	3.721	2	0.156	-
Gender × TTM stages toward regular physical activity	20.368	4	<0.001	0.192
Gender × WTS more on regular physical activity	1.630	2	0.443	-

Source: Authors’ compilation, 2024. Notes: TTM = Transtheoretical model, WTS = Willingness to spend.

**Table 8:** Cross-tabulation of gender and TTM stages toward regular physical activity (N = 550).

			TTM Stages					Total
			1	2	3	4	5	
Gender	male	count	29	52	45	55	83	264
		% within gender	11.0%	19.7%	17.0%	20.8%	31.4%	100.0%
		adjusted standardized residual	2.5	−2.4	−1.5	−1	3.2	
	female	count	15	82	63	70	56	286
		% within gender	5.2%	28.7%	22.0%	24.5%	19.6%	100.0%
		adjusted standardized residual	−2.5	2.4	1.5	1	−3.2	
Total		count	44	134	108	125	139	550
		% within gender	8,0%	24.4%	19.6%	22.7%	25.3%	100.0%

Source: Authors’ compilation, 2024. Notes: TTM = Transtheoretical model, 1 = Precontemplation, 2 = Contemplation, 3 = Preparation, 4 = Action, 5 = Maintenance.

**Table 9:** Relationship between intention to change weight, and TTM stages toward healthy eating and regular physical activity and willingness to spend more on health-promoting foods and regular physical activity (N = 550).

	Chi-Square	df	*p*	Cramer’s V
Intention to Change × TTM stages toward healthy eating	114.627	8	<0.001	0.323
Intention to Change × WTS more on health-promoting foods	5.228	4	0.265	-
Intention to Change × TTM stages toward regular physical activity	56.210	8	<0.001	0.226
Intention to Change × WTS more on regular physical activity	8.248	4	0.083	0.087

Source: Authors’ compilation, 2024. Notes: TTM = Transtheoretical model, WTS = Willingness to spend.

**Table 10:** Cross-tabulation of intention to change weight and TTM stages toward healthy eating (N = 550).

			TTM Stages					Total
			1	2	3	4	5	
Intention to change	want to lose weight	count	23	78	78	72	45	296
		% within gender	7.8%	26.4%	26.4%	24.3%	15.2%	100.0%
		adjusted standardized residual	−8.5	2.4	6.1	3.6	−3.0	
	want to gain weight	count	20	5	4	7	8	44
		% within gender	45.5%	11.4%	9.1%	15.9%	18.2%	100.0%
		adjusted standardized residual	4.0	−1.8	−1.5	−0.5	−0.3	
	satisfied	count	76	40	13	24	57	210
		% within gender	36.2%	19.0%	6.2%	11.4%	27.1%	100.0%
		adjusted standardized residual	6.5	−1.5	−5.4	−3.4	3.3	
Total		count	119	123	95	103	110	550
		% within gender	21,6%	22.4%	17.3%	18.7%	20.0%	100.0%

Source: Authors’ compilation, 2024. Notes: TTM = Transtheoretical model, 1 = Precontemplation, 2 = Contemplation, 3 = Preparation, 4 = Action, 5 = Maintenance.

**Table 11:** Cross-tabulation of intention to change weight and TTM stages toward regular physical activity (N = 550).

			TTM Stages					Total
			1	2	3	4	5	
Intention to change	want to lose weight	count	14	79	72	83	48	296
		% within gender	4.7%	26.7%	24.3%	28.0%	16.2%	100.0%
		adjusted standardized residual	−3.1	1.4	3.0	3.2	−5.3	
	want to gain weight	count	8	8	10	9	9	44
		% within gender	18.2%	18.2%	22.7%	20.5%	20.5%	100.0%
		adjusted standardized residual	2.6	−1.0	0.5	−0.4	−0.8	
	satisfied	count	22	47	26	33	82	210
		% within gender	10.5%	22.4%	12.4%	15.7%	39.0%	100.0%
		adjusted standardized residual	1.7	−0.9	−3.4	−3.1	5.8	
Total		count	44	134	108	125	139	550
		% within gender	8.0%	24.4%	19.6%	22.7%	25.3%	100.0%

Source: Authors’ compilation, 2024. Notes: TTM = Transtheoretical model, 1 = Precontemplation, 2 = Contemplation, 3 = Preparation, 4 = Action, 5 = Maintenance.

## Data Availability

The data used for this study are available and will be shared by the corresponding authors upon request.

## Appendix A

The survey questions
1. Do you think you would need to lose or gain weight to be completely satisfied with your body weight? Choose one option.

- I would need to lose weight
- I would need to gain weight.
- I am satisfied with my body weight.

2. Please describe your eating habits on the following 8-point scale, where 1 means you do not restrict yourself in eating, meaning you eat whatever you want, whenever you want, and 8 means you fully restrict yourself, constantly controlling your food intake and never indulging.

1 = not at all	2	3	4	5	6	7	8 = completely

3. How do you perceive your progress in transitioning to healthier eating?

- I do not intend to transition to a diet I consider healthier within the next six months.
- I feel a strong urge to transition to a diet I consider healthier.
- I will take steps to transition to a diet I consider healthier within the next month.
- I have transitioned to a diet I consider healthier in the past six months.
- I have been eating healthier for more than six months, and the chances of reverting to my old eating habits are minimal.

4. Would you spend more than you currently do on food that benefits your health?

- yes
- no
- I don’t know.

5. How do you perceive your progress in transitioning to regular physical activity?

- I do not intend to transition to what I consider more regular physical activity within the next six months.
- I feel a strong urge to transition to what I consider more regular physical activity.
- I will take steps to transition to what I consider more regular physical activity within the next month.
- I have transitioned to what I consider more regular physical activity in the past six months.-
- I have been engaging in regular physical activity for more than six months, and the chances of reverting to my old habits are minimal.

6. Would you spend more than you currently do on physical activity if it benefited your health?

- yes
- no
- I don’t know.

7. What sources of information do you consult regarding healthy eating, and which do you consider to be authentic?

	Used		Authentic	
	yes	no	yes	no
Local newspaper				
Local radio				
Local television				
National newspaper				
National radio				
National television				
Blogs				
E-newsletters				
Search engine sites				
Social media				
Family members				
Friends and acquaintances				
Dietitians and doctors				
Scientific books				

8. What sources of information do you consult regarding regular physical activity, and which do you consider to be authentic?

	Used		Authentic	
	yes	no	yes	no
Local newspaper				
Local radio				
Local television				
National newspaper				
National radio				
National television				
Blogs				
E-newsletters				
Search engine sites				
Social media				
Family members				
Friends and acquaintances				
Dietitians and doctors				
Scientific books				
Personal trainer				
